# Monte Carlo evaluation of uncertainties in photon and electron TG‐51 absorbed dose calibration

**DOI:** 10.1002/acm2.14339

**Published:** 2024-04-12

**Authors:** Patrick N. McDermott

**Affiliations:** ^1^ Department of Radiation Oncology William Beaumont University Hospital Corewell Health Royal Oak Michigan USA

**Keywords:** absorbed dose calibration, TG‐51

## Abstract

**Purpose:**

The accuracy of dose delivery to all patients treated with medical linacs depends on the accuracy of beam calibration. Dose delivery cannot be any more accurate than this. Given the importance of this, it seems worthwhile taking another look at the expected uncertainty in TG‐51 photon dose calibration and a first look at electron calibration. This work builds on the 2014 addendum to TG‐51 for photons and adds to it by also considering electrons. In that publication, estimates were made of the uncertainty in the dose calibration. In this paper, we take a deeper look at this important issue.

**Methods:**

The methodology used here is more rigorous than previous determinations as it is based on Monte Carlo simulation of uncertainties. It is assumed that mechanical QA has been performed following TG‐142 prior to beam calibration and that there are no uncertainties that exceed the tolerances specified by TG‐142.

**Results/Conclusions:**

Despite the different methodology and assumptions, the estimated uncertainty in photon beam calibration is close to that in the addendum. The careful user should be able to easily reach a 95% confidence interval (CI) of ± 2.3% for photon beam calibration with standard instrumentation. For electron beams calibrated with a Farmer chamber, the estimated uncertainties are slightly larger, and the 95% CI is ±2.6% for 6 MeV and slightly smaller than this for 18 MeV. There is no clear energy dependence in these results. It is unlikely that the user will be able to improve on these uncertainties as the dominant factor in the uncertainty resides in the ion chamber dose calibration factor 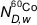. For both photons and electrons, reduction in the ion chamber depth uncertainty below about 0.5 mm and SSD uncertainty below 1 mm have almost no effect on the total dose uncertainty, as uncertainties beyond the user's control totally dominate under these circumstances.

## 1 INTRODUCTION

The accuracy of dose delivery for every patient treated with a linac depends on the accuracy of dose calibration. This makes estimates of dose calibration uncertainty of great importance. The addendum (hereafter addendum) to the TG‐51 absorbed dose calibration protocol has addressed the issue of uncertainties in calibration for photons.^1^, ^2^ The addendum has used a customary statistical formula (Equation 1) to propagate uncertainties. Some of the estimates cited here for input uncertainties differ from the addendum and the methodology for uncertainty propagation is more rigorous. In addition to this, electron beams are considered.

For reference purposes, the standard statistical formula for error propagation is written below. For a quantity $Q=Q(x_{1},x_{2},\text{…},x_{n})$:

(1) $$\sigma_{Q}^{2}\approx \sigma_{x_{1}}^{2}{\left(\frac{\partial Q}{\partial x_{1}}\right)}^{2}+\sigma_{x_{2}}^{2}{\left(\frac{\partial Q}{\partial x_{2}}\right)}^{2}+\cdots +\sigma_{x_{n}}^{2}{\left(\frac{\partial Q}{\partial x_{n}}\right)}^{2},$$

where σ represents the (*k* = 1) standard deviation. This formula is only strictly valid if *Q* is a linear function of all the *x_j_* (*j* = 1,…, *n*) and if *x*_1_… *x_n_* are uncorrelated. As discussed below, some of the values of *x_j_* in the dose calibration calculation are correlated and *Q* is not a linear function of all of the *x_j_* values. It is acknowledged however, that these effects are likely to be small because $\Delta x_{j}/x_{j}\ll 1$. For complex uncertainty propagation problems, it can be more feasible and more accurate to evaluate uncertainties using Monte Carlo simulations. Furthermore, this is relatively easy to accomplish, and the computations are very fast, unlike Monte Carlo simulations of radiation transport.

If all higher order derivatives of *Q* are zero (linear dependence) and if there are no cross correlations, the Monte Carlo results are expected to be the same as standard uncertainty propagation predicts (Equation 1), provided that the number of simulations is sufficiently large. The dose calibration formulas are not totally linear in all input variables and there are cross correlations. As an example of non‐linearity, *R*_50_ appears as an exponent in the equation for $k_{R_{50}}^{\prime }$. Equation (1) predicts that $\sigma_{{k^{\prime }}_{R_{50}}}/{k^{\prime }}_{R_{50}}=1.0\%$ for *R*_50_ = 2.4 cm, whereas Monte Carlo simulation results in 1.6%. As an example of correlation, the quantities *P_ion_* and *P_pol_* are not independent of *M_raw_*, if $M_{raw}^{H}\equiv M_{raw}^{-}\equiv M_{raw}$ as it is implemented by many users of TG‐51.

The uncertainty values in the addendum for *P_ion_* and *P_pol_* appear to be inconsistent with the uncertainty in the “charge measurement,” (0.23% for situation i). If we assume that $M_{raw}^{H}\approx M_{raw}^{L}$, that the uncertainties in these quantities are equal, and that uncertainties combine as given by Equation (1): $\sigma_{P_{ion}}/P_{ion}=\sqrt{2}\sigma_{M_{raw}}/M_{raw}$ (based on the two‐voltage formula). If $\sigma_{M_{raw}}/M_{raw}=0.23\%$ as listed in table II of the addendum, then $\sigma_{P_{ion}}/P_{ion}=0.33\%$. This is considerably larger than the 0.10% listed in table II of the addendum. Furthermore, this assumes that the two‐voltage formula is strictly accurate and that *V_H_* /*V_L_* = 2, exactly.

Some of the estimates of input uncertainties quoted here, differ from those of the addendum. These estimates are based on 20 years of experience with implementation of TG‐51, with both Varian and Elekta linacs using a large variety of instrumentation. The uncertainties quoted here are likely to be similar for other users because the instrumentation used is standard and common, and because the calibration procedure is common to most clinics. The details described are thought to be representative of the cautious clinical user employing standard instrumentation. This is the “simplified” procedure described by the AAPM Working Group Report 374 (“Guidance for TG‐51 reference dosimetry,” hereafter WGTG51) that is believed to be followed by most clinical users.^3^ There is also a discussion of the benefit of the more cautious approach described by WGTG51.

There are two scenarios tabulated in the addendum. Situation (i) assumes “reference class equipment is used.” Situation (ii) makes “typical” assumptions for a “realistic clinical situation.” The scenario adopted here is one in which a clinical user is assumed to be reasonably careful and uses standard clinical instrumentation. It is assumed that the user makes no mistakes resulting in systematic errors. It is wise to have two physicists perform calibration together; the second physicist to check the setup and perform the calculations independently. The monthly constancy check procedure is to be established immediately after calibration. It is essential to have an independent check, such as that provided by the mailed dosimetry service of the Imaging and Radiation Oncology Core (IROC), otherwise the monthly constancy check could simply confirm an erroneous calibration month after month.

Computations of the relative uncertainty in the dose, $\sigma_{D_{w}^{Q}}/D_{w}^{Q}$, have been carried out for specific typical implementations of the TG‐51 protocol for flattened 6 MV and 18 MV photon beams and for 6 MeV and 18 MeV electron beams. The uncertainty analysis is described in the next section by considering the protocol step by step and discussing the uncertainties associated with each step.

## 2 METHODS AND MATERIALS

For Monte Carlo uncertainty propagation, the individual values of a dependent variable $Q=Q(x_{1},x_{2},\text{…},x_{n})$ are calculated as follows:

(2) $$\begin{array}{ccc} Q_{i} & = & Q\left(x_{1}+{\left(\Delta x_{1}\right)}_{i},x_{2}\right.\\ & & \left.+{\left(\Delta x_{2}\right)}_{i},\text{…},x_{n}+{\left(\Delta x_{n}\right)}_{i}\right),\left(i=1,N\right) \end{array}$$

where *N* is a large number and Δ*x_i_* are sampled from a specified distribution with a specified standard deviation. The statistical distributions used for sampling are either a normal (Gaussian) distribution or a uniform distribution, depending on the variable. For a uniform distribution with $x_{-}\leq x_{j}\leq x_{+}$, the standard deviation is:

(3) $$\sigma =\frac{x_{+}-x_{-}}{2\sqrt{3}}.$$

The distribution of the values of *Q_i_* is evaluated by computation of the standard deviation and the 95% confidence interval. Ideally, this simulates repetition of implementation of the protocol *N* times. The value of *N* must be large enough so that repeat computations of the set *Q_i_* result in a standard deviation that does not change in the leading two significant digits. For this problem, it is found that *N* = 10^5^ accomplishes this goal, but *N* = 10^6^ has been used to insure extra stability of the result.

The Monte Carlo simulation was written using the Mathematica (version 12.2) programming language. The code is less than 100 lines long. It has been run on a Core i7 laptop with a clock speed of 2.60 GHz. It typically requires about 1–2 s to complete 10^6^ calculations of $D_{w}^{Q}$. Exploration of uncertainty parameter space is easy because the code runs so quickly.

### 2.1 Photons

This discussion follows the procedure in the TG‐51 protocol step by step. The numbered steps correspond to the numbering in the protocol Worksheet A.

1. **Site data**
  - The linac is an Elekta Versa HD having photon energies of 6, 10, and 18 MV. It is not expected that the analysis for a Varian linac would be appreciably different.
2. **Instrumentation**
  - The Farmer ion chamber is a PTW N30013. This is a waterproof chamber.
  - The electrometer is a Keithley 35040 with *P*_elec_ = 1.000 nC/Rdg and automatic leakage subtraction. The high voltage bias was −300.2 V and the low voltage bias was −150.1 V. Values of *P*_elec_ will be computed as follows:
(4) $${\left(P_{elec}\right)}_{i}=P_{elec}\left(1+{\left(\frac{\Delta P_{elec}}{P_{elec}}\right)}_{i}\right).$$

The calibration uncertainty reported by the ADCL is 0.21%. This is a *k* = 2, 95% level uncertainty. We will therefore sample the 2nd term in Equation (4) from a normal distribution with *σ* = 0.10%.

  c. The ADCL Farmer chamber calibration factor is 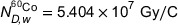, collecting electrode bias +300 V, charge collected is negative. The reported uncertainty (*k* = 2) is 1.4%. Individual values of the calibration factor are computed as follows:

(5)

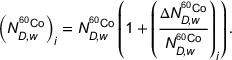

The 2nd term in Equation (5) is sampled from a normal distribution with *σ* = 0.75% (as suggested in table II of the addendum).

3. **Measurement Conditions**
  - Distance

The SSD = 100 cm, field size 10 × 10 cm^2^ at the water surface and 100 MU were delivered for each irradiation.

The gantry angle should be set with a bubble level for best accuracy. According to WGTG51, a 1° error in gantry angle will only result in a 0.0085% error in depth dose at 10 cm deep for 6 MV. The scanning apparatus is leveled using the water phantom water surface itself as a giant level. The ionization chamber should be driven up and down in the water phantom to ensure that the light field cross hair does not move with respect to the chamber. WGTG51 recommends measuring profiles at depths spaced at least 10 cm apart vertically and verifying that the ion chamber is in the center of each profile. Gradients in the beam profiles at the central axis are small for flattened beams. The WGTG51 states that for flattened photon and electron beams, a lateral offset of 2 mm produces dosimetric differences of 0.1% or less at the reference depth. This uncertainty will be neglected for flattened beams. According to WGTG51, for a 10 MV FFF beam, a 1 mm shift in position along the long axis of the Farmer chamber can result in a dosimetric uncertainty of 0.14%.

Although the addendum advises that “the use of a light field distance indicator is not recommended” for setting the SSD, the use of a front pointer or the lasers is problematic. It is difficult to use a front pointer because the surface tension of the water makes it hard to discern when the tip is at the water surface. The use of the lasers is also problematic. Laser position on the sides of the water tank is affected by the degree to which the laser beams are perfectly horizontal even if they point accurately to the isocenter. In addition, meniscus of the water surface at the plastic sides of the water phantom makes the use of the lasers difficult as they cannot be aligned to the water surface at the sides of the tank. The effect of this can amount to 1–2 mm for an acrylic tank. Based on experience it is believed that most users set the SSD to the water surface using the ODI. This is often done by using a thin non‐absorbent material floating on the water surface.^3^ According to Medical Physics Practice Guideline 8, the ODI must be accurate to within 2 mm.^4^ The SSD is therefore sampled from a uniform distribution with a range of ± 2 mm (*σ* = 1.2 mm). No values outside this range are allowed. This corresponds to a standard deviation of 0.11% which compares to the addendum situation (i) value of 0.10%. It is advisable to mark the location of the water surface and the side lasers on the sides of the tank using a piece of masking tape and a marker. This allows a quick check for any change (sagging, evaporation, etc.). The WGTG51 recommends a gantry tilt method for setting the SSD described in appendix A.2 of that document.^3^

  b. Field Size

The field size is set to 10 cm × 10 cm as measured at isocenter. For the Elekta Versa HD, the cross‐plane field size is determined by the MLC when the collimator angle is zero. The TG‐142 report calls for jaw position accuracy for symmetric fields of 2 mm.^5^ The WGTG51 states that the difference between the light field and radiation field can be up to 3 mm when the MLC is used to define the field. It is assumed here that the field size contribution (Δ*f_d_*)*_i_* must fall in the range ± 2 mm (σ = 1.2 mm) with no values outside this range allowed. It is therefore assumed that the distribution is uniform between these limits.

  c. Number of monitor units

The number of monitor units is 100. Variations in the number of MU delivered by the linac from irradiation to irradiation are accounted for below and are included in the statistical variation of *M_raw_* for repeat irradiations with no changes in any settings.

4. **Beam Quality**
  - %dd(10)_x_ = %dd(10), for energy less than 10 MV

The %dd(10) was measured using an IBA CC13 (cavity radius 3 mm) ion chamber with the curve shifted upstream by 0.6 *r_cav_* = 1.8 mm. The uncertainty in the depth will include the effects of the surface setting, uncertainty in the chamber shift correction and the uncertainty in the water phantom ion chamber positioning accuracy. The WGTG51 indicates that the Monte Carlo estimated chamber shift should be 1.2 mm for the CC13 chamber. This differs from 0.6 *r_cav_* by 0.6 mm. The WGTG51 states that the error in the measurement of absorbed dose due to this difference is typically around 0.1% or less.

Uncertainty in %dd(10) is due to uncertainties in the SSD, ion chamber depth and field size. The depth dose is given by:

(6) $$\mathrm{dd}\left(d\right)=\frac{M(d)}{M(d_{m})}\approx {\left(\frac{\mathrm{SSD}+d_{m}}{\mathrm{SSD}+d}\right)}^{2}S_{p}\left(f_{d}\right)\times \mathrm{TMR}\left(d,f_{d}\right),$$

where *d* is the depth, *d_m_* is the depth of maximum dose, *S_p_* is the phantom scatter factor, *f_d_* is the field size at depth (10 cm) and TMR is the tissue maximum ratio. This accounts for inverse square attenuation, attenuation by the water and the effects of field size.

The depth uncertainty depends on setting the ion chamber to the water surface and the accuracy of the water phantom chamber positioning system. It is advisable to use a ruler to test the accuracy of the positioning system for large systematic errors.

The ion chamber is positioned at the water surface by following the reflection technique described in TG‐106.^6^ According to WGTG51, setting the chamber to the water surface using this method can be performed with an uncertainty of 0.5 mm. The positioning accuracy of both the IBA Blue Phantom 2 and the SUN Nuclear 3D SCANNER™ are 0.1 mm.^7^, ^8^ The WGTG51 suggests that the user find the location of the water surface by scanning the ion chamber in small steps through the water surface. It is stated that this method results in an uncertainty of 0.15 mm. Sampling of (Δ*d*)*_i_* is from a uniform distribution with bounds of ± 1 mm (*σ* = 0.58 mm). It is also assumed that (Δ*d_m_*)*_i_* = (Δ*d*)*_i_*.

The uncertainty in the depth dose is given by differentiation of Equation (6):

(7) $$\begin{array}{ccc} {\left(\Delta \mathrm{dd}\right)}_{i} & & \approx \left[\frac{2(d-d_{m})}{\left(\mathrm{SSD}+d\right)\left(\mathrm{SSD}+d_{m}\right)}\right]{\left(\Delta \mathrm{SSD}\right)}_{i}\\ & & +\left[\frac{1}{\mathrm{TMR}}\frac{\partial \mathrm{TMR}}{\partial d}-\frac{2}{\mathrm{SSD}+d}+\frac{2}{\mathrm{SSD}+d_{m}}\right]{\left(\Delta d\right)}_{i}\\ & & +\left[\frac{1}{S_{p}}\frac{\partial S_{p}}{\partial f_{d}}+\frac{1}{\mathrm{TMR}}\frac{\partial \mathrm{TMR}}{\partial f_{d}}\right]{\left(\Delta f_{d}\right)}_{i}, \end{array}$$

where the derivatives are to be evaluated at *d* = 10 cm and *f_d_* = 11 cm. There could be random uncertainties in the charge measurement by the water phantom electrometer that are not accounted for in Equation (7). Any systematic multiplicative uncertainties in the charge measurement will divide out (see Equation (6)). The uncertainty in the depth dominates Equation (7). For 6 MV the coefficient of the ${\left(\Delta d\right)}_{i}$ term is −0.032 cm^−1^ and the coefficients of the ${\left(\Delta \mathrm{SSD}\right)}_{i}$ and ${\left(\Delta f_{d}\right)}_{i}$ terms are 0.0015 cm^−1^ and 0.010 cm^−1^ respectively. The coefficient of the ${\left(\Delta d\right)}_{i}$ term in Equation (7) is in agreement with WGTG51 (≈−0.35%/mm). For 6 MV the calculated standard deviation in %dd(10) is 0.22%.

  b. %dd(10)_x_ for open beams

No lead foil was used, see c. below.

  c. %dd(10)_x_

For energies >10 MV, the “interim” formula (equation 15 of TG‐51) is used to obtain the value of %dd(10)_x_ rather than making a measurement of %dd(10)_Pb_. The interim formula is valid for 75% < %dd(10) < 89% (>45 cm clearance). The value of %dd(10)_x_ is 67.74% for 6 MV and 78.85% for 18 MV.

According to TG‐51 (page 1855) the “interim” formula for %dd(10)_x_ “may cause errors in assigning %dd(10)_x_ of up to 2% in extreme cases.” The values of %dd(10)_x_ will be computed as follows:

(8) $${\left(\%dd{\left(10\right)}_{x}\right)}_{i}=1.267{\left(\%dd(10)\right)}_{i}-20.0+{\left(\Delta dd(10_{x})\right)}_{i},$$

where ${\left(\Delta dd(10_{x})\right)}_{i}$ is sampled from a uniform distribution with limits of ±2% (*σ* = 1.2%).

5. **Determination of *k_Q_***

*k_Q_* is determined from Equation (1) of the addendum, valid for: 63 < %dd(10)_x_ < 86. There are three contributions to the uncertainty in *k_Q_*: the inherent uncertainty in the raw values provided by the protocol document, the uncertainty due to the fit to Equation (1) of the addendum and the propagated uncertainty due to uncertainties in %dd(10)_x_.

(9) $$\begin{array}{ccc} {\left(k_{Q}\right)}_{i} & = & A+B\cdot 10^{-3}\cdot {\left(\%dd{\left(10\right)}_{x}\right)}_{i}+C\cdot 10^{-5}{\left(\%dd{\left(10\right)}_{x}\right)}_{i}^{2}\\ & & +\left[{\left(\frac{\Delta k_{Q}}{k_{Q}}\right)}_{i,0}+{\left(\frac{\Delta k_{Q}}{k_{Q}}\right)}_{i,f}\right]k_{Q}, \end{array}$$

where *A*, *B*, and *C* are constants whose values depend on the specific ion chamber and the first term inside the square brackets represents the sum of the intrinsic uncertainty (subscript 0) and the second term represents the uncertainty from the fit (subscript *f*). For the PTW N30013: *A* = 0.9652, *B *= 2.141, *C* = −2.623. According to the addendum, the average rms deviation associated with the *formula* for *k_Q_* is 0.07%. Therefore, the term ${\left(\Delta k_{Q}/k_{Q}\right)}_{i,f}$ will be sampled from a normal distribution with σ = 0.0007. The inherent uncertainty (Situation i) quoted in the addendum is 0.4%. This is similar to the inherent uncertainty quoted by Andreo (net experimental uncertainty, table 4) of 0.3% for the protocol TRS‐398.^9^ Values of ${\left(\Delta k_{Q}/k_{Q}\right)}_{i,0}$ will be sampled from a normal distribution with *σ* = 0.004.

The calculated standard deviation of *k_Q_* is 0.40% for 6 MV and 0.45% for 18 MV. The absolute deviation of *k_Q_* due to depth alone, Δ*k_Q_*/Δ*d* = −0.00045 mm^−1^, is in agreement with figure A.1.a of WGTG51.^3^ [It is to be noted that for ${\left(\Delta d\right)}_{i}≲1.5\;\mathrm{mm}$, the uncertainty in *k_Q_* is dominated by the uncertainty in the fitting formula.]

6. **Temperature/Pressure Correction**

The ion chamber should be given time to reach thermal equilibrium with the water. It is advisable to use two thermometers with the probes kept in the water. These are usually taped to the side of the water phantom. Fisher NIST traceable Lollipop™ digital thermometers were used that read to the nearest 0.1°C. The water temperature was 20.7°C. Fisher states that the accuracy of these thermometers is ±0.4°C. This uncertainty is larger than recommended by the WGTG51 (±0.3°C). This is a Type B uncertainty. This will be sampled from a uniform distribution with σ_B_ = 0.23°C from Equation (3) following the recommendation of Mitch et al. for handling manufacturer stated uncertainties.^10^ We have six of these thermometers and every six months they are compared to one another. Based on nine separate comparisons, the average standard deviation in the temperature is 0.25°C. This is a Type A uncertainty and it will be sampled from a normal distribution with *σ_A_* = 0.25°C. Individual values of the temperature are given by: $T_{i}\left(C\right)=T_{0}\left(C\right)+\Delta T_{iA}\left(C\right)+\Delta T_{iB}\left(C\right)$, where *T*_0_(C) is the reading of the thermometer.

A mercury barometer was used to measure the atmospheric pressure (742.8 mm‐Hg = 990.1 mbar). Mercury barometers are now rare and therefore the uncertainty analysis shall be based on the reported accuracy of NIST traceable digital barometers. There are a variety of such barometers advertised on the web with stated uncertainties ranging from ±0.3 mbar up to ±8 mbar. The WGTG51 recommends an accuracy of ±0.1 kPa = ±1 mbar. Many of the digital barometers advertised on the web do not meet this criterion. The worst‐case scenario for this type B uncertainty is the ±8 mbar model with σ_B_ = 4.6 mbar or 0.46% (from Equation (3)), to be sampled from a uniform distribution. This is about five times larger than the value 0.09% adopted by Castro et al.^14^ Temperature and pressure uncertainties together lead to an uncertainty (*k* = 1) in *P_TP_* of about 0.48%. The use of a barometer meeting the standards of WGTG51 would reduce this considerably. The addendum cites an uncertainty in *P_TP_* of 0.1% for Situation (i) and 0.4% for Situation (ii).

7. **Polarity Correction**

It is common to use the following relationship during calibration (assuming that the ion chamber was calibrated with the production of negative charge): $M_{raw}^{-}\equiv M_{raw}^{H}\equiv M_{raw}$. In other words, the measurement of *M_raw_* is used in the determination of *P_pol_* and *P_ion_* without separate measurement of $M_{raw}^{H}$ and $M_{raw}^{-}$. It is therefore immediately concluded that ${\left(M_{raw}^{H}\right)}_{i}={\left(M_{raw}^{-}\right)}_{i}={\left(M_{raw}\right)}_{i}$.

It is assumed that the measured charge can be expressed as:

(10) $$\begin{array}{ccc} M_{raw} & & \approx \frac{-k}{{\left(\mathrm{SSD}+d\right)}^{2}}\times S_{c}\left(f\right)\times S_{p}\left(f_{d}\right)\times \mathrm{TMR}\left(d,f_{d}\right)\\ & & \times \;\mathrm{OAR}(r)\times \mathrm{MU}, \end{array}$$

where *k* is a positive constant, *d* is the depth (nominally 10 cm), *S_c_* is the head scatter factor, *S_p_* is the phantom scatter factor, *f* is the field size at isocenter (nominally 10.0 cm), *f_d_* is the field size (nominally 11.0 cm) at depth, OAR is the off‐axis ratio, *r* is the distance off‐axis and MU is the number of monitor units. The minus sign is because we assume negative charge is collected. Off axis effects have been discussed previously. Any uncertainty associated with gantry angle and off axis positioning is neglected for flattened beams.

*M_raw_* may vary from measurement to measurement for repeat irradiations with unchanging setup. That is, without varying the setup in any way, repeat measurements are made. This will be incorporated into the total uncertainty. The contribution for this is represented by a subscript *r*. Such repeat measurements will include any variation in dose delivery by the linac. It is our custom to refrain from discarding the first few measurements as some users do. Our measurements should therefore include the “pre‐irradiation history” effect described in the addendum. Let us look at a typical example. A single electrometer reading for a 6 MV beam under calibration conditions was 12.228 nC. Based on experience (described earlier), this number never varies by more than about 0.005 nC, which is about 0.04%. This Type A variability will be designated ${\left(\Delta M_{raw}/M_{raw}\right)}_{r}$ and it will be sampled from a normal distribution with a standard deviation (*k* = 1) of 0.04%. This is similar to the value adopted by Castro et al of 0.03%.^14^

The addendum includes a correction for leakage current. The electrometers used in most clinics include automatic subtraction of leakage current. It will be assumed that this subtraction is perfect and therefore no correction for this is needed. The addendum states that the leakage term that is used in that publication should include extracameral current and radiation induced leakage such as might originate in an irradiated cable or chamber stem. This is represented by the subscript “xc.” The suggested relative uncertainty for this is 0.1%. This will be sampled from a normal distribution with a standard deviation of 0.1%.

Once the reference chamber is calibrated at the ADCL, the calibration factor may drift. It is assumed here that the clinic does not return the Farmer chamber prematurely for re‐calibration, even if the clinic performs stability or constancy checks. We are not aware of any anecdotal evidence that those clinics that actually perform a stability check return their chambers for recalibration as a result of such a check. In the absence of this, the addendum suggests that the relative stability is in the range 0.3%–0.5%. We adopt a middle value of 0.4%. This will be sampled from a normal distribution with a relative standard deviation of 0.4%. This is indicated by a subscript “st.”

The portion of the uncertainty in the measurement of charge that will be the same for *M_raw_* $M_{raw}^{-}$ and $M_{raw}^{H}$ follows:

(11) $$\begin{array}{c} {\left(\frac{\Delta M_{raw}}{M_{raw}}\right)}_{c,i}=\left(\frac{1}{\mathrm{TMR}}\frac{\partial \mathrm{TMR}}{\partial d}-\frac{2}{\mathrm{SSD}+d}\right){\left(\Delta d\right)}_{i}-\left(\frac{2\;}{\mathrm{SSD}+d}\right){\left(\Delta \mathrm{SSD}\right)}_{i}\\ \;\;+\left(\frac{1}{S_{c,p}}\frac{dS_{c,p}}{df}+\frac{1}{\mathrm{TMR}}\frac{\partial \mathrm{TMR}}{\partial f}\right){\left(\Delta f\right)}_{i}+{\left(\frac{\Delta M_{raw}}{M_{raw}}\right)}_{st,\;i}+{\left(\frac{\Delta M_{raw}}{M_{raw}}\right)}_{xc,\;i} \end{array}$$

The setup uncertainty in depth, SSD and field size, affect all charge measurements: ${\left(\Delta d\right)}_{i}$, ${\left(\Delta \mathrm{SSD}\right)}_{i}$, and ${\left(\Delta f\right)}_{i}$ are the same for all charge measurements. The extracameral contribution is assumed the same for $M_{raw}^{+}$ and $M_{raw}^{L}$. The individual values of the repeatability contribution may be different for $M_{raw}^{+}$ and $M_{raw}^{L}$. For *M_raw_*, we have:

(12) $${\left(\frac{\Delta M_{raw}}{M_{raw}}\right)}_{i}={\left(\frac{\Delta M_{raw}}{M_{raw}}\right)}_{c,\;i}+{\left(\frac{\Delta M_{raw}}{M_{raw}}\right)}_{r,\;i},$$

where the *r* subscript is for repeatability and is sampled from a normal distribution with *σ* = 0.04%.

The value of $M_{raw}^{+}$ is calculated as follows:

(13) $${\left(\Delta M_{raw}^{+}\right)}_{i}={\left[{\left(\frac{\Delta M_{raw}}{M_{raw}}\right)}_{c}+{\left(\frac{\Delta M_{raw}}{M_{raw}}\right)}_{r+}\right]}_{i}M_{raw}^{+},$$

where the second term is drawn from a separate repeatability sampling. The value of *P_pol_* is computed using the usual formula in TG‐51. The calculated standard deviation for *M_raw_* for 6 MV is 0.5% and for *P_pol_* it is 0.03%.

8. ***P_ion_* measurements**

The TG‐51 report states that the accuracy of Equation (12) of the protocol for *P_ion_* is within 0.2% for pulsed beams for a voltage ratio of 2. Let us assume that the voltage ratio, *V_H_* /*V_L_*, is exactly 2, with no uncertainty. Under these optimistic assumptions, uncertainty in *P_ion_* will be due to uncertainty in measurements of $M_{raw}^{H}$ and $M_{raw}^{L}$ plus the uncertainty associated with the use of Equation (12) from TG‐51. In addition to this, *P_ion_* is constrained as follows: 1.00 < *P_ion_* < 1.05. Physically *P_ion_* > 1.00 and the protocol forbids use of an ion chamber for which *P_ion_* > 1.05.

The value of $M_{raw}^{H}$ is calculated as follows:

(14) $${\left(\Delta M_{raw}^{H}\right)}_{i}={\left[{\left(\frac{\Delta M_{raw}}{M_{raw}}\right)}_{c}+{\left(\frac{\Delta M_{raw}}{M_{raw}}\right)}_{rH}\right]}_{i}M_{raw}^{H},$$

where the second term is drawn from a separate repeatability sampling. The equation for (*P_ion_*)*_i_* is:

(15) $${\left(P_{ion}\right)}_{i}=\frac{-1}{{\left(M_{raw}^{H}\right)}_{i}/{\left(M_{raw}^{L}\right)}_{i}-2}+{\left(\frac{\Delta P_{ion}}{P_{ion}}\right)}_{i}P_{ion},$$

where the last term is sampled from a uniform distribution with a range of ±0.002. When (*P_ion_*)*_i_* < 1.0, computed using Equation (15) above, it is set to 1.0. This results in a peak in the statistical frequency distribution of (*P_ion_*)*_i_* values at *P_ion_* = 1.000. For 6 MV the calculated standard deviation in *P_ion_* is 0.2%.

9. **Corrected ion chamber reading**

This is computed using the standard formula (Equation (8) of the TG‐51 protocol).

10. **Dose to water at 10 cm depth**

An uncertainty term due to humidity has been added to the calculation of ${\left(D_{w}^{Q}\right)}_{i}$. We have added a term ${\left(\Delta D_{w}^{Q}/D_{w}^{Q}\right)}_{H}$ for the effects of humidity. According to the addendum this leads to at most a 0.15% uncertainty. We will therefore sample this term from a uniform probability distribution having limits of ±0.15% (*σ* = 0.086%). If clinical normalization is at a different depth than the reference depth, additional uncertainty could be introduced in applying the depth dose to convert to the normalization depth (see WGTG51).

### 2.2 Electrons

This discussion proceeds step by step following TG‐51 Worksheet B. All energies (6–18 MeV) have been calibrated with a Farmer ionization chamber. There was no cross calibration with a plane parallel chamber. The depth‐ionization scans and the measurement of *M_raw_*, and so on, are made separately with a different ionization chamber so that there are no correlations between them (i.e., uncertainties in the depth setting are not the same). The depth‐ionization scans were made with a IBA CC13 ion chamber in an IBA water phantom.

The TG‐51 equation for $D_{w}^{Q}$ contains the product: $M_{raw}\left(d_{ref}\right)P_{gr}^{Q}\left(cyl\right)=M_{raw}\left(d_{ref}\right)\frac{M_{raw}\left(d_{ref}+0.5r_{\mathrm{cav}}\right)}{M_{raw}\left(d_{ref}\right)}=M_{raw}\left(d_{ref}+0.5r_{\mathrm{cav}}\right)$. The value $M_{raw}\left(d_{ref}\right)$ cancels exactly. Therefore, rather than measuring *M_raw_* at two different depths *d_ref_* and *d_ref_* + 0.5 *r_cav_*, the charge is measured only at the latter depth. It is common practice in our clinic to therefore skip the measurement at *d_ref_*. This saves time and may eliminate uncertainties associated with the extra measurement. This presumes that *P*_ion_ and *P*_pol_ have the same value at *d_ref_* and *d_ref_* + 0.5 *r_cav_*.

1. **Site Data**

The linac is an Elekta Versa HD with electron energies of 6, 9, 12, 15, and 18 MeV.

2. **Instrumentation**

The Farmer ion chamber and the electrometer are the same as used for photons.

3. **Measurement Conditions**

The SSD = 100 cm, the applicator is a 10 × 10 cm^2^ applicator and 100 MU are delivered for all charge measurements. It is assumed that there is no uncertainty in the field size as it is determined by the applicator. Uncertainty in the MU delivered is built into the charge measurements as described for photons. Uncertainty in the SSD is the same as described for photons: sampling is from a uniform distribution with a range of ±0.2%.

4. **Beam Quality**

a. All the electron beams have $2\leq I_{50}\leq 10$ cm. *I*_50_ has been measured with an IBA CC13 chamber. The uncertainty in the value of *I*_50_ depends on positioning the scanning ion chamber at the water surface, measuring the depth‐ionization curve and then shifting the curve by 0.5 *r*_cav_ = 1.5 mm. The expected uncertainty in *I*_50_ depends on the depth uncertainty and the uncertainty in the chamber shift. According to WGTG51, the Monte Carlo calculated shift for this chamber should be 0.92 mm. This will lead to a small systematic error in the value of ${k^{\prime }}_{R_{50}}$ of 0.06% for 6 MeV. The expected uncertainty in *I*_50_ is at least as large as the depth uncertainty for the scanning ion chamber but it should be less than the tolerance for energy constancy of the electron beam. According to the AAPM Medical Physics Practice Guidelines for linear accelerator performance, the tolerance for *I*_50_/*R*_50_ constancy measurements is ±2 mm.^11^ It seems reasonable therefore, that *I*_50_ should have an uncertainty of less than 0.15 cm. *I*_50_ is computed by:
(16) $${\left(I_{50}\right)}_{i}=I_{50}+{\left(\Delta I_{50}\right)}_{i},$$

where (Δ*I*_50_)*_i_* is sampled from a uniform distribution with *σ* = 0.087 cm.

b. *R*_50_ is computed from *I*_50_. There are two sources of uncertainty: the uncertainty in *I*_50_ and the uncertainty associated with the formula used to calculate *R*_50_ from *I*_50_. *R*_50_ is computed using a formula from Ding et al. (equation 16 of TG‐51).^12^ This paper states that *R*_50_ “can be estimated to within 0.4 mm.” Individual values of *R*_50_ will be computed as follows:
(17) $${\left[R_{50}\left(\mathrm{cm}\right)\right]}_{i}=1.029{\left(I_{50}\right)}_{i}-0.06+{\left(\Delta R_{50}\right)}_{i},$$

where ${\left(\Delta R_{50}\right)}_{i}$ is sampled from a uniform distribution with limits of ±0.04 cm (*σ* = 0.023 cm).

c. The value of *d_ref_* is computed from: *d_ref _* = 0.6*R*_50_ − 0.1. Individual values of the uncertainty are:
(18) $${\left(\Delta d_{ref}\right)}_{i}=0.6\left[R_{50}-{\left(R_{50}\right)}_{i}\right]+{\left(\Delta d_{set}\right)}_{i},$$

where ${\left(\Delta d_{set}\right)}_{i}$ is the uncertainty in setting the depth of the chamber. As for photons, this is sampled from a uniform distribution with limits of ±0.1 cm.

5. **Determination of *k*_ecal_ and** ${k^{\prime }}_{R_{50}}$

a. *k_ecal_* is determined by consultation of table III of TG‐51. The uncertainty in the value of *k*_ecal_ is unknown to this author. A reasonable assumption is that *k*_ecal_ cannot be determined any more accurately than *k_Q_* for photon beams and therefore we adopt an uncertainty of 0.5%. The value of *k*_ecal_ is computed from:
(19) $${\left(k_{ecal}\right)}_{i}=k_{ecal}+{\left(\Delta k_{ecal}\right)}_{i},$$

where ${\left(\Delta k_{ecal}\right)}_{i}$ is sampled from a normal distribution with *σ* = 0.005 *k*_ecal_.^a^

b. *R*_50_ obeys the inequality $2\leq R_{50}\left(\mathrm{cm}\right)\leq 9$ and we use the analytic expression (equation 19 of TG‐51) for ${k^{\prime }}_{R_{50}}$ for “Farmer‐like cylindrical chambers.” Individual values of ${k^{\prime }}_{R_{50}}$ will be determined as follows:
(20) $$\begin{array}{ccc} {\left({k^{\prime }}_{R_{50}}\right)}_{i} & = & 0.9905+0.071e^{-(R_{50_{i}}/3.67)}\\ & & +{\left(\frac{\Delta {k^{\prime }}_{R_{50}}}{{k^{\prime }}_{R_{50}}}\right)}_{i}{k^{\prime }}_{R_{50}}. \end{array}$$

The last term represents the uncertainty in the fitting formula. TG‐51 states that the maximum error in the formula for ${k^{\prime }}_{R_{50}}$ is 0.2%. We will sample the last term in parentheses from a uniform distribution with an absolute range of ±0.002.

6. **Temperature/Pressure Correction**

a. This is handled in the same way as for photons

7. **Polarity Correction**

Values of $M_{raw}^{+}$ and $M_{raw}^{-}$ are taken from the measurements. The polarity of calibration results in the collection of negative charge (ie. *M_raw_* < 0). It is common to use the following relationship during calibration (assuming that the ion chamber was calibrated with the production of negative charge): $M_{raw}^{-}\equiv M_{raw}^{H}\equiv M_{raw}$. Uncertainties in each of these quantities are therefore identical as discussed previously for photons.

It is assumed that the *M_raw_* can be written as:

(21) $$M_{raw}=K{\left(\frac{\mathrm{VSD}+d_{m}}{\mathrm{VSD}+d_{m}+g}\right)}^{2}\mathrm{DI}\left(d\right),$$

where *K* is a constant, VSD = the virtual source distance, *d_m_* is *d_max_*, *g* is the air gap and DI is the depth‐ionization. We assume that VSD + *d_m_* + *g* is approximately 100 cm and that Δ*g* = ΔSSD. In this case:

(22) $$\begin{array}{ccc} {\left(\Delta M_{raw}\right)}_{c,\;i} & = & \left\{\frac{1}{\mathrm{DI}}\frac{d(\mathrm{DI})}{dd}{\left(\Delta d\right)}_{i}-\left(0.02\right){\left(\Delta \mathrm{SSD}\right)}_{i}\right.\\ & & \left.+{\left(\frac{\Delta M_{raw}}{M_{raw}}\right)}_{st,\;i}+{\left(\frac{\Delta M_{raw}}{M_{raw}}\right)}_{xc,\;i}\right\}M_{raw}, \end{array}$$

where the derivative is evaluated at depth *d_ref_* for the shifted depth‐ionization curve. The depth ionization scans were acquired with an IBA CC13 ionization chamber and an IBA water phantom scanning system and have been shifted by 0.5 *r_cav_*. The derivative has been evaluated by fitting the shifted DI curve to a cubic polynomial in the neighborhood of *d_ref_* and differentiating the polynomial. It is assumed that this uncertainty in depth is the same for all charge measurements as it depends on the initial setup and the ion chamber. The first term in Equation (22) dominates. The second term is on the order of 0.4%. The 3rd term is on the order of 0.3% and the 4th term is on the order of 0.1%. The first term is largest for the lowest energy (6 MeV). For 6 MeV this term is about 0.8% for ${\left(\Delta d\right)}_{i}=1.0\;\mathrm{mm}$. This, by itself, would lead to a 0.8% error in the dose. The first term in Equation (22) declines with increasing energy, dropping by almost a factor of 2 in going from 6 MeV to 18 MeV.

The total value of ${\left(\Delta M_{raw}\right)}_{i}$ is obtained by adding the uncertainty associated with identical irradiations (without changing the setup), as follows:

(23) $${\left(\Delta M_{raw}\left(d_{ref}+0.5r_{cav}\right)\right)}_{i}={\left(\Delta M_{raw}\right)}_{c,\;i}+{\left(\frac{\Delta M_{raw}}{M_{raw}}\right)}_{r,\;i}M_{raw}.$$

The second term in Equation (23) will have different values for (same statistical distribution) $M_{raw}^{+}$ and $M_{raw}^{L}$ as discussed for photons previously. ${\left(\Delta M_{raw}^{+}\right)}_{i}$ and ${\left(\Delta M_{raw}^{L}\right)}_{i}$ are calculated as previously for photons and (*P_pol_*)*_i_* is evaluated using the usual formula. A study of the variations in the electrometer reading for electron beams for repeat identical irradiations shows the value of the repeat contribution to be approximately 0.04%. The calculated standard deviation of *P_pol_* for 6 MeV is about 0.3%.

The WGTG51 states that the shift in the effective point of measurement is 0.38 *r_cav_* for the PTW N30013 ion chamber. If the charge were measured at a depth of *d_ref_* + 0.38 *r_cav_*, instead of *d_ref_* + 0.50 *r_cav_* this would result in a systematic error of roughly 0.2% for 6 MeV. This is estimated from the depth‐ionization curves for this beam.

8. ***P*_ion_ measurements**

The values of (*P_ion_*)*_i_* are calculated using Equation (15) with the constraint that (*P_ion_*)*_i_* > 1.000. It is assumed that *V*_H_/*V*_L_ = 2 (exactly). The calculated standard deviation of *P_ion_* is about 0.2% for 6 MeV.

9. **Corrected ion chamber reading *M***

a. This is computed using the TG‐51 formula. *M* is evaluated at depth *d_ref_* + 0.5 *r_cav_* as explained above.

10. **Dose to water at reference depth, *d_ref_***

a. This is computed using the TG‐51 formula. It is handled the same way as the computation for photons (step 9 for photons) and includes uncertainty due to humidity.

## 3 RESULTS

### 3.1 Photons

The uncertainty budget for 6 MV is shown in Table 1. The third column “uncertainty level” represents assigned uncertainties for various quantities as discussed above. The last column indicates the contribution to $\sigma_{D_{w}^{Q}}/D_{w}^{Q}$ from the quantities specified in the rows. These values were calculated by setting all other uncertainties to zero. The shaded rows in Table 1 represent uncertainties that are not under the control of the clinical user.

**TABLE 1:** Uncertainty budget for 6 MV (Flattened) beam calibration.^a^

Quantity	Symbol	Uncertainty Level σ	Sampling distribution	Contribution to $\sigma_{D_{w}^{Q}}/D_{w}^{Q}$ (%)
SSD of water surface	SSD	0.12 cm	Uniform distribution ΔSSD ≤ ± 0.2 cm	0.21
Depth of Farmer chamber	*d*	0.058 cm	Uniform distribution Δ*d* ≤ ± 0.1 cm	0.30
Temperature	*T* (°C)	0.25°C (Type A) 0.23°C (Type B)	Normal Gaussian Uniform distribution	0.12
Pressure	*p*	4.6 mbar	Uniform distribution	0.47
Field size	*f*	0.12 cm	Uniform distribution Δ*f* ≤ ± 0.2 cm	0.10
*k_Q_ intrinsic*	*k_Q_*	0.4%	Normal Gaussian	0.40
*k_Q_ formula* ^b^	*k_Q_*	0.07%	Normal Gaussian	0.071
Repeat irradiations of Farmer chamber	${\left(\Delta M_{raw}/M_{raw}\right)}_{r}$	0.04%	Normal Gaussian	0.075
Stability of ion chamber calibration	${\left(\Delta M_{raw}/M_{raw}\right)}_{\mathrm{st}}$	0.4%	Normal Gaussian	0.40
Extracameral current	${\left(\Delta M_{raw}/M_{raw}\right)}_{\mathrm{xc}}$	0.1%	Normal Gaussian	0.10
Electrometer calibration	*P*_elec_	0.1%	Normal Gaussian	0.10
Ion chamber calibration factor	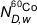	0.75%	Normal Gaussian	0.75
Two voltage *formula* for *P_ion_* ^b^	*P_ion_*	0.2%	Normal Gaussian, but *P_ion_* ≥ 1.000	0.18
Humidity	——	0.087%	Uniform distribution 0.15% maximum	0.087
		Total absorbed dose $\sigma_{D_{w}^{Q}}/D_{w}^{Q}$		**1.2**

^a^ Shaded rows list quantities (mostly) outside the user's control. An anonymous reviewer suggests that the uncertainty associated with stability of the ion chamber calibration and repeat irradiations of the Farmer chamber are not completely outside user control. Ion chamber stability can be monitored and repeat irradiation is influenced by factors such as linac warm‐up, and so on.

^b^ This is the uncertainty for the fitting formula only and does not include propagated uncertainties.

The *k* = 1 uncertainty in the absorbed dose for 6 MV is 1.15% given the assumptions in §II. The 95% confidence interval for 6 MV is therefore 2.3%. Scenario (ii) of the addendum, with a CI of 95% = 4.2%, would appear to be possible only if the user is recklessly careless. The statistical distribution in the values of ${\left(D_{w}^{Q}\right)}_{i}$ is a normal distribution. The histogram of this distribution of 10^6^ values is indistinguishable from the graph of a normal distribution having the same mean and standard deviation. For 18 MV, the uncertainty is almost the same as for 6 MV (1.16%). IROC finds no energy dependence in its OSLD system audits of the ratio (IROC measured dose)/ (institution reported dose) for photons. IROC reports the standard deviation in this ratio to be 1.5% for 1772 measurements.^13^

It does not seem that these uncertainties are likely to differ much for other users because the instrumentation used is very common and the choices in carrying out the protocol are also common. Interestingly, the uncertainty calculated here for photons is about the same as quoted in the addendum for scenario (i) (0.9%) for *reference class instrumentation*. This suggests that the addendum may be overly pessimistic about the level of uncertainty that can be achieved by the *careful user in a realistic clinical setting*. Castro et al. have analyzed the uncertainty budget for the IAEA TRS‐398 protocol and find the (*k* = 1) uncertainty to be 1.3%.^14^

Given that $\sigma_{D_{w}^{Q}}/D_{w}^{Q}=1.15\%$, the probability of measuring a dose outside the limits ±5% is on the order of 1 in 10^5^. Therefore, any TG‐51 measured dose falling outside these limits, is almost surely due to an error. Consider however, an independent assessment of the dose using OSLD having *σ* = 1.7% as reported by Kry et al. for IROC.^16^ The *k* = 1 uncertainty of the ratio of the doses, *D*_OSLD_ */D*_TG51_, will be 2.05%. In this case, the probability that the dose ratio will fall outside ±5% is1.5%. IROC reports about 0.5% of photon beams outside this tolerance.^16^

The user has control over SSD, *d*, *T*, *p*, and *f*. The remainder of the uncertainties are mostly beyond user control. If all user uncertainties are set to zero $\sigma_{D_{w}^{Q}}/D_{w}^{Q}=0.975\%$ for 6 MV. This indicates that the careful user has little opportunity for reduction in the uncertainty. This implies that even if all the rigorous recommendations of WGTG51 are followed, that at most, the reduction in the 95% CI would be 0.35%. The largest contribution to the uncertainty, by far, resides in the calibration factor 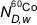.

**FIGURE 1:**
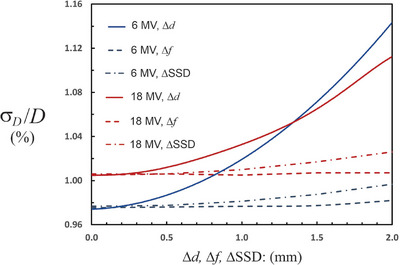
The relative uncertainty in the dose for 6 MV and 18 MV photon beams as a function of Δ*d*, Δ*f* and ΔSSD. Uncertainties outside the user's control are held fixed as listed in Table 1 (rows highlighted in grey). The Δ’s are sampled from a uniform distribution with the limits shown on the horizontal axis. For Δ*d* < 0.5 mm there is almost no improvement in the dose uncertainty. The same is true when the Δ*f* and the ΔSSD are less than 2 mm. The uncertainty in the dose is dominated by factors outside the user's control under these circumstances.

Figure 1 shows a graph of the relative standard deviation in the dose for both 6 MV and 18 MV as a function of Δ*d*, ΔSSD, and Δ*f*. These input quantities were sampled from uniform distributions. For each plot, all other user‐controlled uncertainties are set to zero. All the non‐user uncertainties retain the values shown in Table 1 (shaded rows). The plots show that there is almost no uncertainty benefit to determining the depth to an accuracy better than 0.5 mm. This is because the depth uncertainty is overwhelmed by other uncertainties beyond the user's control. Efforts to reduce the uncertainty in the depth to a level of 0.15 mm (WGTG51) are unlikely to yield any benefit. For the SSD and the field size, there is no uncertainty benefit to determination with an uncertainty less than 2 mm. This situation could change if the uncertainty in the quantities beyond the users control are reduced significantly, particularly the uncertainty in 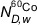.

### 3.2 Electrons

The uncertainty budget for 6 MeV electrons is shown in Table 2. Given the input uncertainties in Table 2, the *k* = 1 uncertainty is 1.3% (95% CI is 2.6%) for 6 MeV. For 18 MeV the 95% CI is slightly smaller at 2.4%. Intermediate electron energies show similar uncertainties and thus there is no pronounced energy dependence. Elbashir et al. have compared the results of electron beam dose calibration using three different protocols: IAEA TRS398, TG‐51 and DIN 6800‐2.^15^ For cylindrical chambers these authors report an uncertainty (*k* = 1) of 1.4%. Castro et al. report the *k* = 1 uncertainty for electrons calibrated with a plane parallel Markus chamber using the IAEA TRS‐398 protocol as1.7%.^10^ IROC shows a somewhat larger standard deviation for the ratio (IROC/institution) for electrons (all energies, measured with OSLD) than for photons (1.8% vs. 1.5%).^8^ Kry et al. report that there were “significantly more” IROC results outside of a 5% tolerance for electron beams than for photon beams based on remote audits using OSLD.^16^ These authors observed a possible energy dependence with about 1.2% of 6 MeV beams outside a 5% tolerance and almost 1.5% of 18 MeV beams outside this tolerance. Intermediate energies were all less than 1% outside this tolerance. For the uncertainty budget reported in Table 2, it is expected that only about 1 in 8000 6 MeV beams should fall outside a ±5% tolerance if the discrepancies are only due to random uncertainties. Consider however, an independent assessment of the dose using OSLD having *σ* = 1.7%. The *k* = 1 uncertainty of the ratio of the doses, *D*_OSLD_ */D*_TG51_, will be 2.14%. In this case the probability that the dose ratio will fall outside ±5% is 1.9% for 6 MeV.

**TABLE 2:** Uncertainty budget for 6 MeV beam calibration.^a^

Quantity	Symbol	Uncertainty level σ	Sampling distribution	Contribution to $\sigma_{D_{w}^{Q}}/D_{w}^{Q}$ (%)
SSD of water surface	SSD	0.12 cm	Uniform distribution ΔSSD ≤ ± 0.2 cm	0.23
Depth of Farmer chamber	*d*	0.058 cm	Uniform distribution Δ*d* ≤ ± 0.1 cm	0.47
Temperature	*T* (°C)	0.25°C (Type A) 0.23°C (Type B)	Normal Gaussian Uniform distribution	0.12
Pressure	*p*	4.6 mbar	Uniform distribution	0.47
depth of 50% ionization	*I*_50_	0.087 cm	Uniform distribution Δ*I*_50_ ≤ 0.15 cm	0.35
Identical irradiations of Farmer chamber	${\left(\Delta M_{raw}/M_{raw}\right)}_{B}$	0.04%	Normal Gaussian	0.075
Stability of ion chamber calibration	${\left(\Delta M_{raw}/M_{raw}\right)}_{\mathrm{st}}$	0.3%	Normal Gaussian	0.30
Extracameral current	${\left(\Delta M_{raw}/M_{raw}\right)}_{\mathrm{xc}}$	0.10%	Normal Gaussian	0.10
Electrometer calibration	*P*_elec_	0.10%	Normal Gaussian	0.10
Ion chamber calibration factor	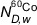	0.75%	Normal Gaussian	0.75
Formula for *R*_50_ ^b^	*R*_50_	0.023 cm	Uniform distribution Δ*R*_50_ ≤ 0.04 cm	0.27
Beam quality	*k*_ecal_	0.005	Normal Gaussian	0.50
Beam quality fitting formula ^b^	${k^{\prime }}_{R_{50}}$	0.12%	Uniform distribution $\Delta {k^{\prime }}_{R_{50}}/{k^{\prime }}_{R_{50}}\leq 0.2\%$	0.11
Two voltage *formula* for *P_ion_* ^b^	*P_ion_*	0.2%	Normal Gaussian, but *P_ion_* ≥ 1.000	0.18
Humidity	——	0.087%	Uniform distribution 0.15% maximum	0.087
		Total Absorbed Dose $\sigma_{D_{w}^{Q}}/D_{w}^{Q}$		**1.3**

^a^ Shaded rows contain quantities that are (mostly) outside the user's control.

^b^ This is the uncertainty for the fitting formula only and does not include propagated uncertainties from parameters that are set or measured.

**FIGURE 2:**
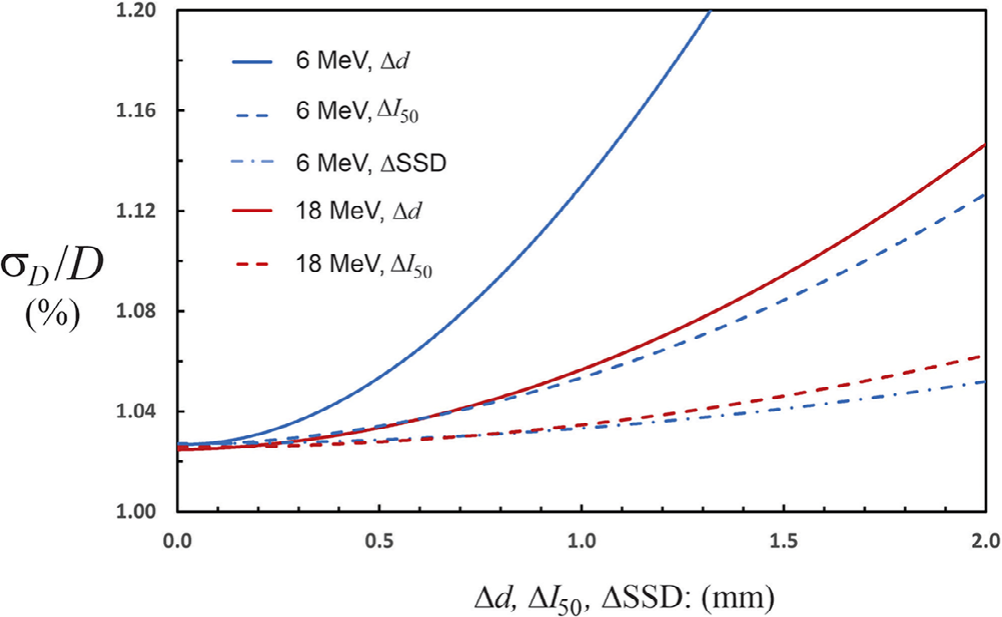
The relative uncertainty in the dose for 6 MeV and 18 MeV electron beams as a function of Δ*d*, Δ*I*_50_ and ΔSSD. Uncertainties outside the user's control are held fixed as listed in Table 2 (rows highlighted in grey). The Δ’s are sampled from a uniform distribution with the limits shown on the horizontal axis. For Δ*d* < 0.5 mm there is almost no improvement in the dose uncertainty. The same is true when the Δ*I*_50_ and the ΔSSD are less than 1 mm. The uncertainty in the dose is dominated by factors outside the user's control under these circumstances.

The major user‐controlled contributors to the uncertainty are the depth *d* and *I*_50_. For 6 MeV, if all the user controlled uncertainties are set to zero (temp, pressure, depth, SSD, and *I*_50_), the 95% CI becomes 2.0%. This shows that the careful user cannot significantly reduce the uncertainty. Figure 2 shows a graph of the relative standard deviation in the dose for both 6 MeV and 18 MeV as a function of Δ*d*, ΔSSD, and Δ*I*_50_. The input quantities were sampled from uniform distributions. For each graph, all other user‐controlled uncertainties are set to zero. All the non‐user uncertainties retain the values shown in Table 2 (shaded rows). The graphs show that there is almost no uncertainty benefit to determining the depth to an accuracy better than 0.5 mm. This is because the depth uncertainty is overwhelmed by other uncertainties beyond the user's control. For the SSD and the field size, there is little dose uncertainty reduction for uncertainties less than 2 mm.

## 4 CONCLUSION

Uncertainties in absolute dose calibration for 6 MV and 18 MV photons and for 6 MeV and 18 MeV electron beams have been estimated using Monte Carlo simulation of 10^6^ calibrations. Each run of 10^6^ calibrations requires 1–2 s of CPU time. It should be reasonably easy to reach a 95% CI of ±2.3% for photon beam calibration and ±2.6% for electron beam calibration. There is no pronounced energy dependence for these results. Each user of TG‐51 must carefully evaluate their own uncertainties, which may differ from those presented here. It is thought, however, that the uncertainties quoted here are representative of the results for the careful user employing common commercially available instrumentation. The addendum to TG‐51 is, if anything, overly pessimistic about the level of uncertainty that can be achieved for photon dose calibration by the careful typical clinical user employing standard instrumentation. There are no easy steps that the user can take to improve on these uncertainties even if following the rigorous approach described in the Working Group report on TG‐51. The majority of the contribution to the uncertainty is out of the users’ control.

The philosophy to be adopted here should be borrowed from radiation safety: ALARA—as low as reasonably achievable. It is unlikely that going beyond the “simple” methods discussed in WGTG51 to the more complicated methods discussed therein will yield any significant improvement in dose calibration uncertainty. However, some of the less simple methods discussed by the working group, especially those that are relatively quick and easy to implement, will serve at least as a check on the simplified methods and may help to avoid mistakes leading to systematic errors.

## CONFLICT OF INTEREST STATEMENT

The authors declare no conflicts of interest.

## Footnotes

a An anonymous reviewer has suggested two references for the uncertainty in *k_ecal_*: Mainegra et al.^17^ and Muir et al.^18^ Neither of these publications explicitly state uncertainties in *k_ecal_* but both publications “suggest that an uncertainty of 0.5% is not unreasonable, at least for cylindrical chambers.”
